# Complete Genome Sequence of Bluetongue Virus Serotype 1 Circulating in Italy, Obtained through a Fast Next-Generation Sequencing Protocol

**DOI:** 10.1128/genomeA.00093-14

**Published:** 2014-02-13

**Authors:** Alessio Lorusso, Maurilia Marcacci, Massimo Ancora, Iolanda Mangone, Alessandra Leone, Valeria Marini, Cesare Cammà, Giovanni Savini

**Affiliations:** OIE Reference Laboratory for Bluetongue, Istituto Zooprofilattico Sperimentale dell’Abruzzo e Molise G. Caporale, Teramo, Italy

## Abstract

A field strain of the bluetongue virus serotype 1 (BTV-1) was isolated from infected sheep in Sardinia, Italy, in October 2013. The genome was sequenced using Ion Torrent technology. BTV-1 strain SAD2013 belongs to the Western topotype of BTV-1, clustering with BTV-1 strains isolated in Europe and northern Africa since 2006.

## GENOME ANNOUNCEMENT

The constellation of the bluetongue virus (BTV) genome may vary depending on the occurrence of reassortment events (1), just like with influenza viruses (2). Thus, the heterogeneity of the BTV genome poses the need to consider innovative techniques devoted to sequencing the whole genome in a reasonably short time. The analysis of all genome segments is crucial when a novel BT outbreak occurs in a given area, even more so when multiple serotypes circulate at the same time or when modified live vaccines have been used in the past in the outbreak area. BTV serotype 1 (BTV-1) reoccurred in Sardinia, Italy, in 2012 (3) and 2013. Unlike in 2012, in 2013, BTV-1 moved toward Corsica, Sicily, and mainland Italy. We describe the complete genome sequence of BTV-1 SAD2013 obtained through an innovative and fast Ion Torrent-based next-generation sequencing (NGS) protocol. Viral double-stranded RNA (dsRNA) was purified (4), and reverse transcription was conducted in the presence of 50 ng of random hexamers and 0.5 ng of primers specific to the ends of the BTV genome segments (5′-GTTAAAN-3′ and 5′-GTAAGTN-3′) (5). Double-stranded cDNA was prepared using the second-strand cDNA synthesis kit (New England BioLabs) and employed for enzymatic fragmentation and adapter ligation using the Ion Plus fragment library kit (Life Technologies). Size selection was performed, followed by quantification on a bioanalyzer. For template preparation, the library was hybridized to the Ion Sphere particles (ISPs) in a process that involves emulsion PCR, bead breaking, and enrichment (Ion OneTouch 200 template kit version 2 DL). The enriched ISPs were loaded onto the Ion 314 Chip and sequenced in the Ion PGM platform. The sequencing run delivered 173.6 Mb of sequence data. In total, we acquired 505,150 reads of BTV-1 SAD2013, ranging from 8 to 373 bp in length. All low-quality bases were trimmed from the sequence reads, and the remaining reads were *de novo* assembled using MIRA version 4.0rc4, which yielded a total of 3,633 contigs with 22.1% coverage. It is worthwhile to stress that the whole genome of BTV-1 SAD2013 was sequenced and analyzed in fewer than three working days, starting from the purification of viral dsRNA from cell culture. The BTV-1 SAD2013 genome was demonstrated to be nearly identical to those of BTV-1 FRA2007/18 (GenBank accession no. JX861487 to JX861496), isolated in France, BTV-1 SAD2012, isolated in Sardinia in 2012, and the partially sequenced BTV-1 strains isolated in northern Africa and Europe since 2006. The paramount duties of the OIE Reference Laboratory for Bluetongue of Teramo are to perform molecular epidemiological analyses of BTV and the related orbiviruses and to immediately share genomic data on public databases. From this perspective, the assessment of fast and robust NGS protocols for a dsRNA genome, such as the one described in this paper, is pivotal. Furthermore, the full-length sequence of BTV-1 SAD2013 may serve in the synthesis of reverse genetics plasmids.

### Nucleotide sequence accession numbers.

The nucleotide sequences for BTV-1 SAD2013 have been deposited in GenBank under accession no. KJ019205 to KJ019214.
